# The NAPRESSIM trial: the use of low-dose, prophylactic naloxone infusion to prevent respiratory depression with intrathecally administered morphine in elective hepatobiliary surgery: a study protocol and statistical analysis plan for a randomised controlled trial

**DOI:** 10.1186/s13063-017-2370-0

**Published:** 2017-12-29

**Authors:** David Cosgrave, Marie Galligan, Era Soukhin, Victoria McMullan, Siobhan McGuinness, Anand Puttappa, Niamh Conlon, John Boylan, Rabia Hussain, Peter Doran, Alistair Nichol

**Affiliations:** 1 St Vincent’s University Hospital, Dublin, Ireland; 2 School of Medicine, University College Dublin, Dublin, Ireland; 3 Monash University, Melbourne, VIC Australia; 4 The Alfred Hospital, Melbourne, VIC Australia

**Keywords:** Intrathecal morphine, Naloxone, Respiratory depression, Randomised controlled trials

## Abstract

**Background:**

Intrathecally administered morphine is effective as part of a postoperative analgesia regimen following major hepatopancreaticobiliary surgery. However, the potential for postoperative respiratory depression at the doses required for effective analgesia currently limits its clinical use. The use of a low-dose, prophylactic naloxone infusion following intrathecally administered morphine may significantly reduce postoperative respiratory depression. The NAPRESSIM trial aims to answer this question.

**Methods/design:**

‘The use of low-dose, prophylactic naloxone infusion to prevent respiratory depression with intrathecally administered morphine’ trial is an investigator-led, single-centre, randomised, double-blind, placebo-controlled, double-arm comparator study. The trial will recruit 96 patients aged > 18 years, undergoing major open hepatopancreaticobiliary resections, who are receiving intrathecally administered morphine as part of a standard anaesthetic regimen. It aims to investigate whether the prophylactic administration of naloxone via intravenous infusion compared to placebo will reduce the proportion of episodes of respiratory depression in this cohort of patients.

Trial patients will receive an infusion of naloxone or placebo, commenced within 1 h of postoperative extubation continued until the first postoperative morning. The primary outcome is the rate of respiratory depression in the intervention group as compared to the placebo group. Secondary outcomes include pain scores, rates of nausea and vomiting, pruritus, sedation scores and adverse outcomes. We will also employ a novel, non-invasive, respiratory minute volume monitor (ExSpiron 1Xi, Respiratory Motion, Inc., 411 Waverley Oaks Road, Building 1, Suite 150, Waltham, MA, USA) to assess the monitor’s accuracy for detecting respiratory depression.

**Discussion:**

The trial aims to provide a clear management plan to prevent respiratory depression after the intrathecal administration of morphine, and thereby improve patient safety.

**Trial registration:**

ClinicalTrials.gov, ID: NCT02885948. Registered retrospectively on 4 July 2016.

Protocol Version 2.0, 3 April 2017.

Protocol identification (code or reference number): UCDCRC/15/006

EudraCT registration number: 2015-003504-22. Registered on 5 August 2015.

**Electronic supplementary material:**

The online version of this article (doi:10.1186/s13063-017-2370-0) contains supplementary material, which is available to authorized users.

## Background

Liver, pancreatic and biliary resections are common surgical procedures worldwide. However, the optimum modality of analgesia remains controversial [1–4]. Some centres utilise thoracic epidural analgesia (TEA) as part of their postoperative analgesic regimen [5–8] and the use of TEA has been advocated as improving outcomes, providing superior analgesia and increasing recurrence-free survival in patients undergoing resection of colorectal liver metastases [1, 7, 9–14]. However, others have raised concerns [15–17], namely: the theoretical risk of epidural haematoma in the setting of increased risk of coagulopathy, increased intravenously administered fluid volume associated with TEA, prolonged immobility, failure of analgesia [18] and increased length of stay [19] compared to available alternative methods of analgesia. Although the incidence of epidural haematoma is rare [20], mild-to-moderate coagulation abnormalities after major liver resection are not uncommon [21–23], and in this setting it is reasonable to assume an increased risk of haematoma. Coagulation abnormalities also affect timing of removal of the epidural catheter in patients post liver resection [17]. Some components of enhanced recovery programmes, such as early mobilisation and strict fluid balance, can be adversely affected by epidural analgesia [24, 25].

A number of studies have shown the benefit of intrathecally administered morphine (ITM) in this patient cohort [26–31]. The dose remains debatable, but the requirement for higher doses of ITM than in obstetric or pelvic procedures has been shown [32–34]. Unfortunately, at the doses required (up to 1 mg has been studied and shown to be effective), the risk of respiratory depression is well documented [35–37]. Rates of respiratory depression are variable in the academic literature (between 0.36% and 7.0%) [38–44], but there are methodological variances which may explain the difference in reported rates; also, there are few studies of homogenous patient groups. Even the definition of respiratory depression remains controversial [45, 46]. There is some consensus that patients receiving doses of ITM over 300 mcg should be monitored at a level above general ward level care, in order to monitor for, and treat, respiratory depression without any adverse outcome for the patient [47, 48]. The majority of hepatopancreaticobiliary anaesthetists in our centre routinely use a combination of ITM at a dose of 10 mcg/kg, administered prior to induction of anaesthesia, abdominal wall local anaesthetic blocks (transversus abdominus plane blocks and rectus sheath blocks) administered after induction under ultrasound guidance, and surgically positioned wound infiltration catheters placed at the end of surgery.

Apart from respiratory depression, the risk of nausea and vomiting, pruritus and urinary retention are significant following ITM [35, 49]. While not life-threatening, these complications are upsetting for patients and are considered to be significant events in the postoperative period [49, 50].

One suggested method of reducing respiratory depression and other side effects is the use of a prophylactic naloxone infusion. Naloxone has been reported in the academic literature as a reversal agent for morphine-induced respiratory depression since 1973 [51]. However, the bulk of publications refer to its use as a treatment rather than a prophylactic agent. Some publications address prophylaxis of respiratory depression, but available academic literature is mainly restricted to the obstetric setting [52, 53]. Two retrospective analyses in a urological surgery cohort (*N* = 35) and a gynaecological surgery cohort (*N* = 98) of patients have been published [54, 55] showing a good safety profile for the combination of higher doses of ITM with prophylactic naloxone infusion. These papers did not compare to a placebo group. No prospective data exist using this regimen in our study population.

Our study aims to investigate whether a similar prophylactic naloxone infusion in a cohort of patients undergoing major open hepatopancreaticobiliary resections with ITM will reduce the incidence of respiratory depression (primary) and other clinically significant side effects (secondary).

## Methods/design

### Trial design

This is an investigator-led, single-centre, randomised, double-blind, placebo-controlled, double-arm comparator study. It aims to investigate whether the prophylactic administration of naloxone via intravenous infusion compared to placebo will reduce the risk of respiratory depression to a clinically significant degree. The trial will recruit 96 patients aged 18 years or older, undergoing major open hepatopancreaticobiliary resections who will receive ITM 10 mcg/kg as part of a standard anaesthetic regimen, in St Vincent’s University Hospital, Dublin, Ireland.

### Participants

**Fig. 1:**
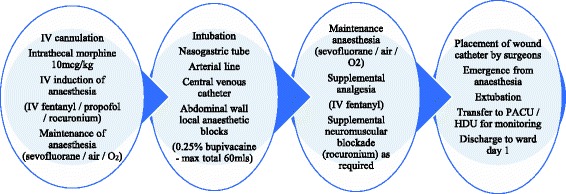
Standard anaesthetic management. Schematic diagram showing standard anaesthetic management of open hepatobiliary surgical patients in our unit

Participants will be those patients undergoing any major open hepatopancreaticobiliary resection in our university hospital hepatopancreaticobiliary surgery unit. Recruited patients will undergo surgery with standard anaesthetic management as shown in Fig. 1. They will also have an additional non-invasive monitor, the ExSpiron 1Xi (Respiratory Motion, Inc., 411 Waverley Oaks Road, Building 1, Suite 150, Waltham, MA, USA) applied. This will be used for data collection only, and no clinical decisions will be made based on its readings.

### Inclusion and exclusion criteria

Patients who undergo major open hepatopancreaticobiliary resections and receive ITM 10 mcg/kg as part of their standard anaesthetic management. See Table 1.

**Table 1:** Eligibility criteria for participants

Inclusion criteria	Exclusion criteria
> 18 years old at screening	Allergy/sensitivity to naloxone
Presenting for elective major open hepatopancreaticobiliary resection under general anaesthetic	Female subjects who are pregnant or breast feeding
Able/willing to give written informed consent and to comply with the requirements of the study	Received any other investigational medicine within the prior 2 months
	Receiving anticonvulsant medications
	Cardiac arrhythmia with uncontrolled rate
	History of chronic opioid use/chronic pain
	Contraindication to intrathecal injection
	Documented history of obstructive sleep apnoea
	Treating clinician does not have equipoise to randomise this patient into the study

### Setting

The trial will be carried out in the hepatopancreaticobiliary surgery unit of St Vincent’s University Hospital. Participants will be cared for in either the post-anaesthesia care unit (PACU) or the Bloomfield high-dependency unit (HDU) for the duration of the trial period. Nursing care will be at least a ratio of 1:2 for the duration of the trial. All data collected pertaining to the primary and secondary outcomes will be collected as part of the routine postoperative nursing care documentation.

### Ethical approval, funding and study sponsorship

This study was approved by the St Vincent’s University Hospital Research Ethics Committee and the Health Products Regulatory Authority of Ireland. Both bodies must be informed in writing of any changes to the protocol, prior to any such changes being implemented. The Study was funded by an unrestricted grant from the St Vincent’s Anaesthetic Foundation. The study was supported by the Irish Critical Care-Clinical Trials Core methodology hub in the Clinical Research Centre, University College Dublin. The Clinical Research Centre in University College Dublin acted as the sponsor for this study. Neither the sponsor, nor any of the study funders, will have any involvement in data collection, data review, data analysis or preparation of the publication manuscript or in the decision to submit for publication.

### Outcome measures

The primary outcome of this trial is the rate of respiratory depression during the study period for patients receiving naloxone infusion compared to placebo. Respiratory depression will be diagnosed based mainly on criteria derived from the American Society of Anesthesiologist’s (ASA) ‘Practice Guidelines for the Prevention, Detection and Management of Respiratory Depression Associated with Neuraxial Opioid Administration’ [40] 2016 document. The criteria we will use are: respiratory rate < 10 and/or a decrease in SpO_2_ to < 92% and/or an increase in FiO_2_ required to maintain SpO_2_ > 92%. The primary outcome will be recorded as a dichotomous variable, recording any episode of respiratory depression during administration of the study infusion. Study infusion will be administered from within 60 min of extubation until 8:00 a.m. on the first postoperative morning.

### Secondary outcomes

Secondary outcomes will be recorded as the occurrence of the dichotomous variables presence or absence of pruritus and nausea/vomiting at any time over the monitoring period, and continuous outcomes will be assessed over the same time period.

1. Incidence of pruritus
  Presence or absence of pruritus will be recorded on an hourly basis, along with the need for any rescue medication for treatment of pruritus
2. Incidence of nausea and vomiting
  Patients will be asked to report any incidence of nausea on an hourly basis and any episode of vomiting will be recorded. The use of any antiemetic agents, the frequency of administration and the total dose required will be recorded
3. Ramsey Sedation Score
  The Ramsey Sedation Score will be recorded on an hourly basis. Any change in sedation score in the treatment arm will be assessed
4. Pain scores
  We use a numerical rating scale from 0 to 10 in assessing pain in our unit, and this will be recorded on an hourly basis
5. Requirement for supplemental fentanyl (rescue analgesia)
  Total dose of rescue intravenously administered fentanyl administered during the study period will be assessed
6. Patient overall satisfaction with analgesia
  We will utilise a simple Visual Analogue Scale, which will be scored at the end of the study period, by the investigator
7. Rate of respiratory depression associated with a Ramsey Sedation Score ≥ 3
  We will assess the respiratory depression parameters listed above, combined with Ramsey Sedation Scores
8. Rate of respiratory depression associated with a PaCO_2_ ≥ 50 mmHg
  We will assess the respiratory depression parameters, combined with the 2-hourly PaCO_2_ results
9. Any differences in haemodynamic parameters between study groups
  We will compare heart rate (HR), systolic and diastolic blood pressures and need for any vasopressor support between the two groups
10. Major adverse events, adverse events and protocol deviations will be recorded and compared between the two groups

### Exploratory objective

To determine the accuracy of the Respiratory Motion ExSpiron 1Xi versus clinical observations in detecting respiratory depression in our patient population, we will collect the minute-volume data, and note all episodes of respiratory depression compared to the traditional diagnostic parameters listed above. We will also evaluate the relationship between minute volume and PaCO_2_ to determine if changes in minute volume are reflected by changes in PaCO_2_ levels. In view of the inaccuracy of clinical observations in diagnosing respiratory depression, this may add to the diagnostic armoury for improving diagnosis.

- Exploratory outcomes: minute volume
  - ○ Respiratory depression as diagnosed by ExSpiron manufacturer criteria – minute volume decreased to 40% predicted

### Interventions

**Fig. 2:**
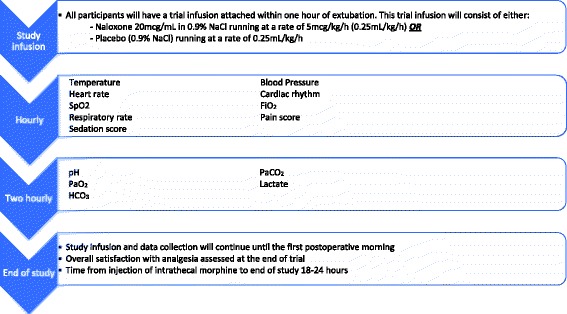
Data collection. Schematic diagram demonstrating the data which will be collected, and the timing of that data collection. *All observations may be collected more frequently if clinically indicated

Intervention in trial participants will include standard anaesthetic management as per Fig. 1, plus the addition of a study infusion postoperatively. The study infusion is an intravenous infusion of naloxone hydrochloride 20 mcg/mL, administered at a rate of 0.25 mL/kg/h (5 mcg/kg/h) or placebo (0.9% NaCl) at 0.25 mL/kg/h. Postoperative follow-up will be the same in both arms of the trial. Figure 2 shows the frequency and type of data collected. Patients will be monitored for 18 to 24 h post administration of ITM, exceeding the maximum risk period for respiratory depression. Study involvement ends at 8:00 a.m. the morning after surgery. All patients will have ITM administered prior to 2:00 pm on the day of surgery, meaning that the duration of monitoring in all patients is at least 18 h post administration of ITM, as is standard practice in our unit.

### Investigational product

Naloxone, the study medication, will be supplied to the St Vincent’s University Hospital pharmacy by Mercury Pharma Ltd. (Dublin, Ireland). Each 1-mL ampoule of solution contains 400 mcg (0.4 mg) naloxone hydrochloride present as naloxone hydrochloride dihydrate. This will be diluted to a concentration of 20 mcg/mL with 0.9% NaCl and presented as a clear, colourless, sterile solution for infusion. The study infusion is for clinical trial use only and can only be administered to the patient named on the drug label. The study infusion will be commenced within 1 h of extubating the patient and continued until 8:00 a.m. the morning after surgery. Unused study solution can be disposed of by nursing staff, in compliance with standard practice for disposing of unused medications/intravenously administered fluids. Placebo is 0.9% NaCl. Both the active infusion and placebo will be prepared according to the randomisation schedule in the hospital sterile isolator department.

### Screening and consent

Theatre lists will be reviewed in advance of the day of surgery to assess for patients having eligible surgical procedures. These patients will then be screened either on the ward or in the pre-operative assessment clinic in order to assess if the individual patient meets eligibility criteria. The patient will be provided with a patient information leaflet and allowed time to read through it. On the morning of surgery the patient will be given the opportunity to ask questions, prior to discussing and signing consent.

### Randomisation

Trial treatment will be allocated between study drug and placebo in a ratio of 1:1 according to variable block randomisation, with block sizes of 4 and 6. The randomisation schedule will be drawn up by a trial statistician using the statistical software R (R Core Team, 2016). The randomisation schedule will consist of a list of randomisation codes, each with a corresponding treatment assignment.

Randomisation will be implemented by the study pharmacist. All eligible consenting patients will be randomised on the morning of surgery. The treating clinician will assign each patient a randomisation code and will inform the study pharmacist of this code. The study pharmacist will use the pre-prepared randomisation schedule to determine treatment allocation of a patient (study drug or placebo) according to the patient’s randomisation code. They will not inform the treating physician, nursing staff or allocated patient.

### Blinding

The study will be conducted as a blinded trial. Patients, treating physicians and nursing staff will be unaware of the treatment allocations. To maintain blinding of these trial team members, some of the trial staff will be unblinded; specifically, the study pharmacist and the trial statistician.

The study pharmacist will prepare either the study drug or the placebo solution, according to the randomisation schedule. The infusions will be prepared in the aseptic unit on the morning of surgery and will be dispatched to the PACU to be commenced within 1 h of extubation. Both study drug and placebo solutions will appear identical: clear in colour, packaged and labelled identically by the trial pharmacist.

Procedures will be put in place for emergency unblinding. The blinding can be broken in the event of a serious adverse event (SAE) or at request of the treating clinician to the study principal investigator (PI), due to clinical need. In the event of an allergic reaction where the allergen is unclear, even though the incidence of anaphylactic reaction to naloxone is rare, the study infusion should be stopped immediately and the treatment allocation of the patient will be unblinded.

For the purposes of emergency unblinding, a copy of the randomisation schedule will be stored in sequentially numbered opaque sealed envelopes (labelled with randomisation codes), in a cupboard in the administration office of the intensive care unit (ICU), in very close proximity to PACU. The PI and ICU nursing managers will be aware of the location of the randomisation schedule in the event of an emergency.

The study PI will know the location of the subject randomisation codes and will be contacted in any emergency situation to assist with unblinding the allocation. The contact details for the PI will be recorded in the clinical notes and on the anaesthetic sheet in the clinical notes. Nursing staff will be made aware of the location of these contact details during handover of the patient from the operating theatre.

When final database entries have been made and final queries have been resolved, the study database will be locked. A blind review of the data will take place, prior to final data analysis. For the blind review, the study statistician will receive the study dataset with the absence of patient randomisation codes, and with treatment arms labelled but not identified. The purpose of the blind review will be to assess data for protocol violations, to check for outliers, to determine whether variable transformations are appropriate and to decide whether the planned statistical analysis is appropriate. The blind review will inform the final plan for analysis.

#### Data management

Data as outlined above will be collected by the on-site investigators from the postoperative care records. This will be recorded on a data sheet, and transferred to an electronic Case Report Form (CRF). Data reported on the CRF that are derived from source documents must be consistent with the source documents or the discrepancies must be explained. The clinical study monitor and representatives of the regulatory authority can directly access source documents for comparison of data therein with the data in the electronic CRFs and can verify that the study is carried out in compliance with the protocol and local regulatory requirements.

The full dataset should be recorded for all enrolled patients. The investigators will adhere to national and hospital protocols on data use and storage. All paper records will be kept in a locked file cabinet. Research participant data will be stored also on a dedicated, protected research electronic database. The information entered into the electronic database will be stored by the sponsor responsible for the CRF. This server has managed access and password protection. All reads and writes to the database will be recorded with date, time and user. Users will have a unique internal identification and data entry will require an electronic signature which consists of system password and username. A quality assurance audit may be conducted by the sponsor or its agent at any time during, or shortly after, the study.

#### Confidentiality

The trial staff will ensure that the subjects’ anonymity is maintained. The subjects will be identified only by initials and a subject’s identification number on the CRF and any database. All documents will be stored securely. The study will comply with the Data Protection Act.

### Statistical analysis plan

A Consolidated Standard of Reporting Trials (CONSORT) flow diagram will be generated to illustrate the flow of patients through the trial. This will show the number of patients reviewed for eligibility, how many were ineligible, reasons for ineligibility, the number consented, the number randomised and treated and the differences between groups. It will also show the number of patients with adverse events, protocol deviations and any losses to follow-up. The total number of patients analysed in each group will also be shown.

Baseline data, as well as primary and secondary outcomes will be summarised by treatment arm, using descriptive statistics. The following process variables will also be summarised by treatment group using descriptive statistics and compared between treatment groups using *t* tests (or appropriate non-parametric test):

- Duration of follow-up
- Duration of surgery
- Duration of study infusion
- Time from injection of ITM to end of study
- Dose of intraoperative intravenously administered fentanyl

For inferential analysis of primary and secondary outcomes, treatment effect will be considered statistically significant if the obtained *p* value is less than 0.05.

The main statistical analyses of primary and secondary outcomes will be conducted following the modified intention-to-treat (ITT) principle on a full analysis set of patients, excluding those who withdrew consent to participate or have their data used in the study. A further per-protocol analysis will be carried out on all primary and secondary outcomes for patients who received any dose of the study infusion.

### Sample size and power

Sample size was estimated to determine the superiority of the prophylactic naloxone infusion compared to placebo in reducing the rate of respiratory depression. Based on an audit of 29 patient charts in our unit containing information on respiratory depression, the percentage of the target patient population who develop respiratory depression without treatment is estimated to be 31% (95% confidence interval (CI) 14.2–47.9%). This is based on the definition of respiratory depression in the ASA guidelines for prevention of respiratory depression with ITM [40].

A clinically significant difference was defined as a 75% reduction in the percentage of respiratory depression among those on treatment, compared to those on placebo. Given the estimate of 31% for the placebo group, this is equivalent to a clinically significant relative risk of 4 (a four-times greater risk of respiratory depression for patients on placebo, than for those on treatment).

Sample size calculations are based on a superiority hypothesis test of the risk of respiratory depression, formulated with the objective of testing whether the risk of respiratory depression is less for those on treatment than for those on placebo. To achieve a power of 80% for this test at a 5% significance level, a sample size of 43 per group is required or a total sample size of 86. This sample size calculation was calculated based on a one-tailed, two-proportion Z test with continuity correction.

After commencement of the study the sample size was inflated to compensate for an un-anticipated dropout rate of up to 10%. The final sample size is 96 (48 per treatment group).

### Primary outcome analysis

The primary outcome will be summarised as the number and percentage of patients in each treatment arm who develop respiratory depression within the study period. Percentages will be calculated using the number of patients for whom data are available as the denominator. Denominators will also be reported.

Risk of respiratory depression will be compared across treatment arms using a risk ratio with an associated 95% CI.

The primary analysis will test the superiority hypothesis that the risk of respiratory depression is reduced for patients receiving a prophylactic naloxone infusion, compared to patients receiving a placebo infusion. The primary outcome will be modelled as a binomial random variable, using a one-sided, two-proportion Z test, with continuity correction.

Sensitivity analysis of the primary outcome will be conducted using logistic regression modelling, adjusting for potentially relevant covariates, to determine whether the estimated treatment effect differs after covariate adjustment. Potentially relevant covariates to be considered include:

- Age
- BMI (Body Mass Index), Duration of surgery
- Duration of study infusion
- Time from injection of ITM to end of study
- Dose of intraoperative intravenously administered fentanyl
- Dose of postoperative intravenously administered fentanyl

### Secondary outcome analysis

Maximum pain score in 24 h will be calculated for each patient. Descriptive statistics for maximum pain score will be calculated and compared across treatment groups. A Mann-Whitney *U* test will test the hypothesis of a difference in the distribution of maximum pain scores between naloxone and placebo.

Line charts will be used to display hourly pain scores over a 24-h period, by treatment group, and to identify differences in patterns of pain over time between treatment groups. Pain scores over time will be modelled using a generalised mixed-effects model, with an appropriate link function, in order to test for differences in patterns of pain over time for naloxone and placebo. This model will be adjusted for relevant covariates (as for the primary outcome) to determine sensitivity of pain score patterns to these covariates.

Requirement for supplemental fentanyl (mcg) will be summarised by treatment group with appropriate summary statistics. A *t* test, or equivalent non-parametric alternative where appropriate, will test the hypothesis of a difference in the mean requirement for supplemental fentanyl between naloxone and placebo. Sensitivity analysis will be conducted using linear regression modelling, adjusting for relevant covariates (as for the primary outcome), to determine whether the estimated treatment effect differs after covariate adjustment.

Scores on patient satisfaction with mode of analgesia by treatment group. A Mann-Whitney *U* test will test the hypothesis of a difference in the distribution of scores of patient satisfaction with analgesia between naloxone and placebo.

Binary secondary outcomes include: presence of absence of pruritus, presence or absence of nausea, and/or presence or absence of vomiting, any Ramsey Sedation Score ≥ 3 and any PaCO_2_ > 6.66 kPa. Each binary secondary outcome will be summarised by treatment group, using frequencies and percentages. Ratios of relative risk will be presented with 95% CIs, for comparison of naloxone and placebo groups. Odds ratios with 95% CIs will also be calculated and presented in supplementary material. For each binary secondary outcome, a two-sided Z test will test the hypothesis of a difference in the outcome between naloxone and placebo. Sensitivity analyses will be conducted for binary secondary outcomes with logistic regression models, adjusting for relevant covariates (as listed above for the primary outcome) to determine whether the estimated treatment effect differs after adjustment for these covariates.

#### Exploratory outcomes analysis

For the Respiratory Motion ExSpiron 1Xi, respiratory depression will be defined as per the manufacturers recommendations: minute volume decreased to 40% predicted. Sensitivity, specificity, positive predictive value and negative predictive value of the Respiratory Motion ExSpiron 1Xi for the diagnosis of respiratory depression will then be calculated (with 95% CIs) with reference to the ‘gold standard’ of clinical observation. For patients diagnosed with respiratory depression, we will also evaluate the correlation between the change in minute volume at time of diagnosis with changes in PaCO_2_ at the same time point. The relationship over time between PaCO_2_ and minute volume will be evaluated using mixed-effects regression models fitted to data from all patients (regardless of respiratory depression status).

#### Safety monitoring

The site monitor will regularly review data on adverse events, protocol deviations, and any occurrence of SAEs. However, we have not employed a data monitoring committee, as the sample size is small, and the intervention is low risk. We are confident that by monitoring adverse events through the sponsor, any safety issues which arise will be addressed promptly. Data on safety and tolerability will be summarised by treatment group using descriptive statistical methods, with 95% CIs where appropriate. Patients with adverse events and/or protocol deviations will be identified and evaluated, using a descriptive analysis, to summarise their baseline characteristics and their treatment experience. All patients who received any amount of study infusion (naloxone or placebo) will be included in the safety analysis.

### Publication of results

The authors intend to publish the results of this trial in a high-quality, peer-reviewed journal upon completion of data collection and analysis.

## Discussion

Intrathecally administered morphine is an effective component of multimodal analgesia for hepatopancreaticobiliary surgical patients. However, the risk of respiratory depression necessitates a higher monitoring workload than would otherwise be necessary. The administration of a prophylactic naloxone infusion has the potential to reduce this risk, making ITM a potentially viable and safer option for patients (Additional file 1).

### Current status

The NAPRESSIM trial commenced in April 2016 at St Vincent’s University Hospital, Dublin, Ireland. Recruitment is proceeding and the aim is to achieve the target sample size on or before December 2017.

## Additional file

Additional file 1: SPIRIT 2013 Checklist: recommended items to address in a clinical trial protocol and related documents*. (DOC 122 kb)

## Abbreviations

ASA: American Society of Anesthesiologists
BMI: Body Mass Index
CI: Confidence interval
CONSORT: Consolidated Standards of Reporting Trials
CRF: Case Report Form
eCRF: electronic Case Report Form
HDU: High-dependency unit
HR: Heart rate
ICU: Intensive care unit
ITM: Intrathecally administered morphine
ITT: Intention-to-treat
IV: Intravenous
NaCl: Sodium chloride
PACU: Post-anaesthesia care unit
PI: Principal investigator
SAE: Serious adverse event
TEA: Thoracic epidural analgesia
